# Patellar Tendon Excision and Repair for Residual Patella Alta after Prior Failed Patellar Tendon Repair: Surgical Decision Making and Outcome

**DOI:** 10.1155/2018/7964732

**Published:** 2018-07-24

**Authors:** Richard N. Puzzitiello, Avinesh Agarwalla, Austin Stone, Brian Forsythe

**Affiliations:** Midwest Orthopaedics at Rush, Rush University Medical Center, Chicago, IL, USA

## Abstract

Presented in this report is a complex revision case of a patellar tendon repair preceded by excess tendon excision to correct for recurrent patella alta deformity, in a workers' compensation patient. The goal of this procedure was to alleviate this patient's pain, to preserve his ability to function in his activities of daily living, and to allow him to return to work at some capacity. On postoperative radiographs, the revision procedure appeared to have successfully corrected this patient's patella alta deformity. After an extended rehabilitation process, this patient had reached maximal medical improvement at 1-year follow-up. He displayed modest improvements in all PROs, including a clinically significant improvement in his short-form mental component score. Despite his functional capacity being still somewhat limited, this patient reported subjective satisfaction after this complicated salvage procedure.

## 1. Introduction

Patellar tendon rupture is an uncommon yet disabling injury most frequently seen in active adults under 40 years old. Surgical intervention is necessary to restore extensor mechanism functionality [1]. Several surgical techniques and rehabilitation protocols can be utilized to address this pathology based on the mechanism and chronicity of the injury [1]. Outcomes following patellar tendon repair have shown high levels of patient satisfaction and low rates of complications [2–5]. Patella alta, a common radiographic sign associated with patellar tendon rupture, has previously been reported to persist in isolated cases and is considered a surgical failure [5]. Strategies for a failed patellar tendon repair are limited in the literature.

In this report, we present the case of a workers' compensation patient who received a revision procedure for recurrence of symptoms and evidence of patella alta seven months following primary nonaugmented repair of a patellar tendon rupture with early postoperative mobilization. This patient received a patellar tendon advancement procedure by excision and repair of the patellar tendon using two PEEK corkscrew anchors fixed to the patella. This is a unique case of a salvage procedure with modest outcomes following a previously undescribed procedure for a known complication after patellar tendon repair.

## 2. Case Report

**Figure 1:**
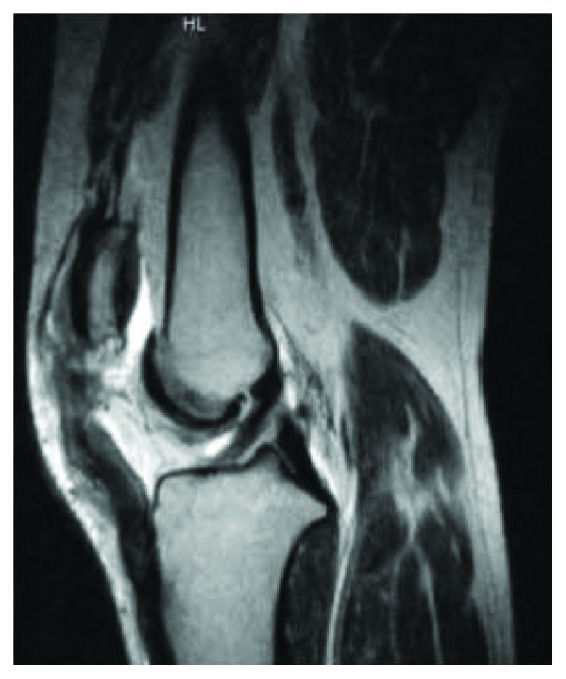
T2-weighted MRI of the right knee demonstrating patella alta and a full thickness tear of his proximal patellar tendon, two months following injury and one month prior to this patient's primary procedure. Insall-Salvati Index = 1.4, Caton-Deschamps Index = 1.34.

**Figure 2:**
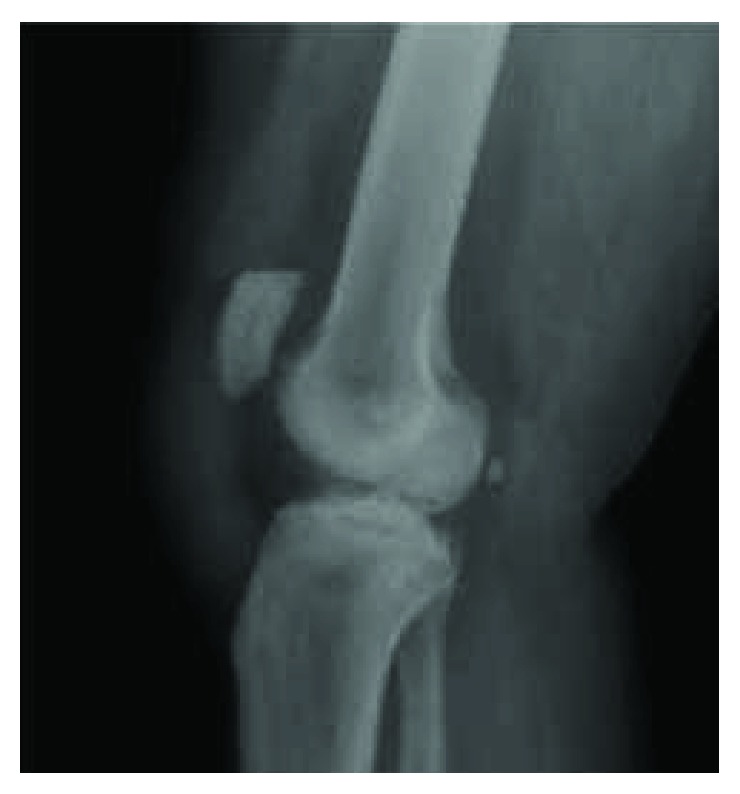
Postoperative lateral X-ray of the patient's right knee demonstrating a corrected patella alta deformity immediately following the first procedure. Insall-Salvati Index = 1.23, Caton-Deschamps Index (CDI) = 1.14.

**Figure 3:**
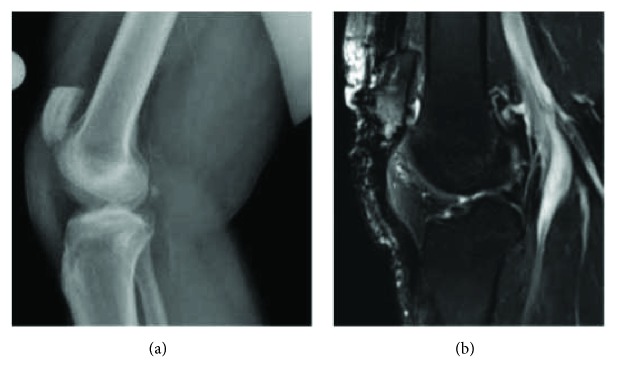
Lateral X-ray (a) and T2-weighted MRI (b) of the patient's right knee eleven months following the index procedure showing proximal migration of the patella and a redundant but intact patellar tendon. CDI = 1.51, ISI = 1.55.

A 45-year-old male sustained a traumatic work-related patellar tendon rupture from the inferior pole of the patella while exiting a vehicle. The patient had a past medical history of diabetes mellitus type II. The patient was evaluated within 22 days of his injury and initially treated with primary repair 81 days after the injury. The tendon was repaired with two number 2 nonabsorbable sutures in a Krackow suture configuration throughout the length of the patellar tendon and anchored through bone tunnels in the patella. This patellar height was corrected to an Insall-Salvati Index (ISI) and Caton-Deschamps Index (CDI) of 1.23 and 1.14 (Figure 1) from 1.4 and 1.34, respectively (Figure 2). His knee was immobilized in a locking brace for two weeks, and then physical therapy was initiated for range of motion at two weeks postoperatively. The patient progressed slowly through physical therapy gaining 100 degrees of active leg flexion but developed significant quadriceps atrophy, patella alta, and 10 degrees of an extensor lag at 7 months following the procedure. The patient was compliant with the standard rehabilitation protocol and had no history of traumatic reinjury. Eleven months after the primary procedure, the patient was referred to our clinic for persistent pain, pain with squatting and kneeling, instability, and stagnation in functional recovery which prevented him from returning to work up to this point. Subjectively, he reported a 4/10 pain level at rest. Clinical examination revealed proximal migration of the patella, 2+ coarse patellar crepitus, full active range of motion, 3+/5 quadriceps strength, and a 10-degree lag with single leg raise. T2-weighted MRI and lateral knee radiograph at 11-month follow-up confirmed patella alta deformity (CDI = 1.51, ISI = 1.55), an intact albeit lax patellar tendon, and cartilage fissuring near the inferior patellar apex (Figure 3). There was no additional ligamentous injury noted on MRI. His preoperative patient-reported outcome scores can be found in Table 1. A collective decision was made with the patient at this time to proceed with revision patellar tendon repair with the goal of returning to work at some capacity and resuming his normal activities of daily living.

**Figure 4:**
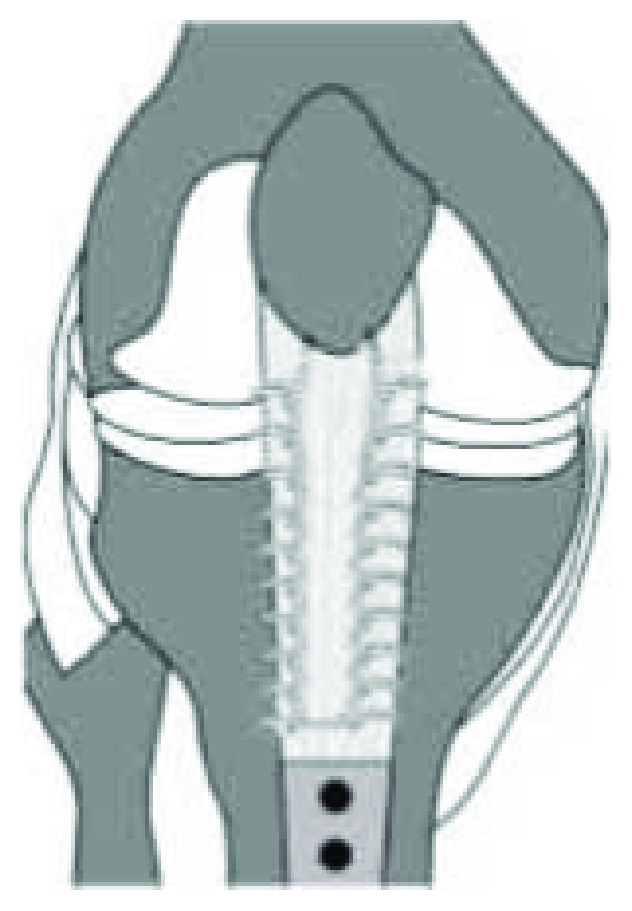
Schematic demonstrating the anchor placement and stitching technique used to fixate the patellar tendon repair.

**Figure 5:**
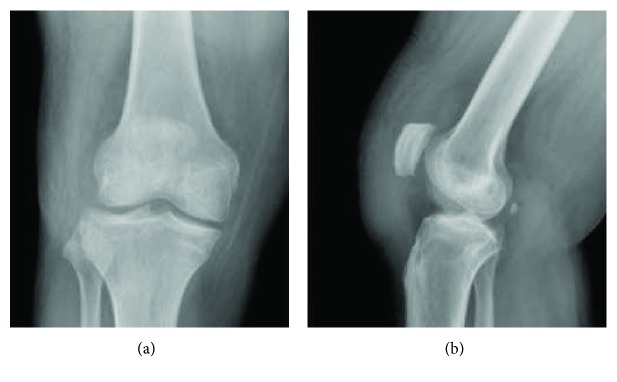
Postoperative AP (a) and lateral (b) view of the right knee displaying a corrected patellar height after the revision procedure. Caton-Deschamps Index = 1.09, Insall-Salvati Index = 1.16.

**Figure 6:**
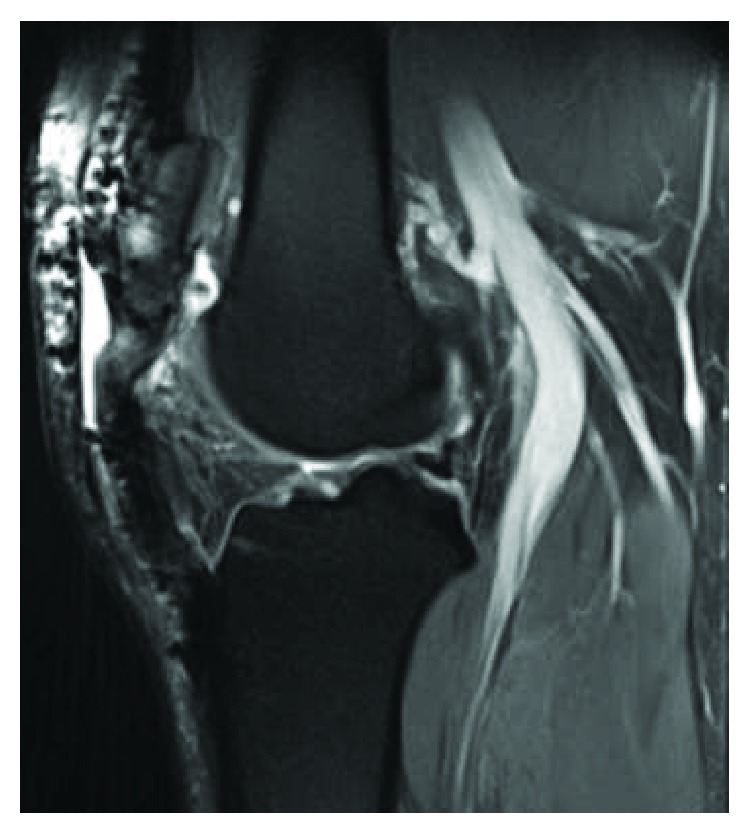
Postoperative MRI of the right knee at 18-month follow-up. Caton-Deschamps = 1.35.

The previous midline incision was dissected to visualize and confirm obvious redundancy and thinning of the patellar tendon. A 2 × 4 × 1 cm rectangular block of redundant patellar tendon tissue was outlined and resected to correct the degree of patella alta. The patella was mobilized using blunt dissection. The suture material from the index repair was removed, and the distal patellar footprint was prepared. Two 2.5 PEEK corkscrew anchors (Arthrex, Naples, FL) were anchored 2 cm apart on the distal patellar footprint. Krackow sutures were passed through the midsubstance of the patellar tendon, and with the opposite limb of each stitch, a half hitch was made such that a pulley mechanism was created (Figure 4). The tendon was reapproximated, and final fixation was secured with four mattress anchor knots with five alternating half hitches. The knee was brought to 30 degrees of flexion and the construct was stable. The wound was closed with a standard layered closure. Postoperative lateral knee radiograph displayed a CDI of 1.09 and an ISI of 1.16 (Figure 5), which confirmed that patella alta had been corrected. At 18-month follow-up, the patient had a repeat MRI performed which demonstrated a CDI of 1.35 (Figure 6).

**Figure 7:**
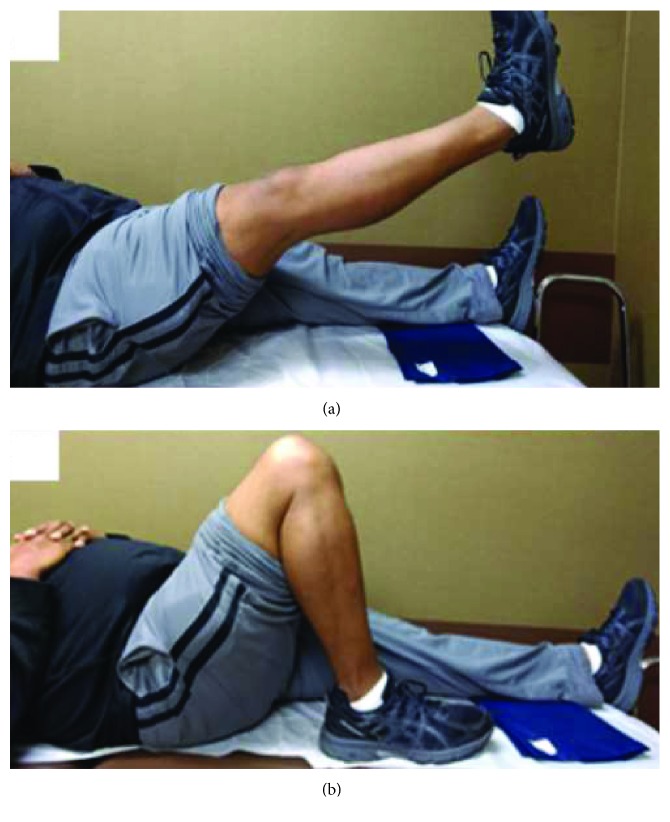
Postoperative examination of the patient's active range of motion at 1-year follow-up. (a) Patients' surgical leg in full extension. (b) Patients' leg in 135 degrees of flexion.

Following the operation, a locked extension brace was applied for full-time use, and he began physical therapy two weeks postoperatively. The patient was compliant with his rehabilitation protocol and did not suffer any setbacks in the postoperative period. At his 1-year follow-up examination, the patient did not appear in acute distress, had no joint effusion, did not have patellar apprehension, demonstrated a nonantalgic gait, showed full active range of motion with no lag (Figure 7), displayed a negative Clarke exam, and had 4/5 quadriceps strength. In addition, the patient had 5 mm of anterior translation bilaterally on KT-1000 arthrometry testing and 20.6 pounds of force on maximal muscle testing of leg extension on the right compared to 21.3 pounds on the left as measured by a handheld dynamometer. The patient reported a pain level of 2/10 with activity, which was a decrease from his preoperative pain level (4/10). He reported occasional use of Tylenol for pain. His PRO scores at 1-year follow-up can be found in Table 1. Despite a relatively benign physical exam (Figure 7) and subjective reporting of satisfaction with the revision procedure and his outcome, the patient reported moderate functional limitations. Permanent work-duty restrictions were subsequently outlined as a functional capacity examination, and patient reported outcomes did not permit return to his full occupational capacity.

## 3. Discussion

Presented in this report is a complex revision case of a patellar tendon repair, with the goal of alleviating this patient's pain and to preserve his ability to function in his activities of daily living. After an extended rehabilitation process, this patient had reached maximal medical improvement resulting in modest improvements in all PROs and ability to return to work albeit with permanent functional restrictions.

Although there are no previously defined values for the minimal clinically important difference (MCID) for PROs after patellar tendon repair, extrapolating the MCIDs for ACL reconstruction [6], this patient demonstrated that he did have an improvement in his SF-12 mental component score that reached clinical significance. The SF-12 mental component score is a general health-related quality of life which gives an assessment of a patients' well-being and has previously been shown to be reflective of the relative success of surgery [6]. Although this patient had moderate functional limitations at maximal medical improvement, clinically, this patient appreciated a subjective improvement in his general well-being. In addition, this patient had many risk factors predisposing him to worse outcomes including low preoperative functioning, workers' compensation status [7, 8], history of type 2 diabetes [9, 10], and obesity [11] (BMI = 37.5). The only other cases of a revision patellar tendon repair identified in the literature resulted in full functional recoveries; however, these patients were 27 and 28 years old, and both were professional athletes [12, 13].

Historically, the treatment of choice for acute patellar tendon rupture has been primary repair augmented with cerclage wire, suture, or grafting to bridge the repair, followed by extended periods (≥6 weeks) of immobilization [2, 3]. However, these techniques have been associated with pain, weakness, patella baja, and decreased mobility with high rates of arthrofibrosis [14, 15]. In addition, augmentation with cables, wires, and Mersilene tape is frequently symptomatic and often requires a second surgery for implant removal [16]. A distinct advantage of primary repair with nonabsorbable suture is that it allows early mobilization and physical therapy initiation within one week of surgery and does not necessitate a second procedure to remove material [15]. Previous reports of primary repair with nonabsorbable suture have shown good functional and objective outcomes, with as much as an 85% return to preinjury level in patients receiving this procedure [14, 15]. For these reasons, primary repair utilizing nonabsorbable sutures to treat this patient's acute patellar tendon injury was decided to be utilized. An important factor taken into consideration was the potential for gap formation with which was previously demonstrated in biomechanical studies of this procedure choice [17, 18]. However, it was ultimately decided that the potential benefits outweighed the risks for this procedure in comparison to the other techniques for patellar tendon repair.

Patella alta, defined as a CDI > 1.2 [19], creates higher patellofemoral contact forces, which causes anterior knee pain and may limit functionality in patients [20]. The primary technique used to correct patella alta is a tibial tubercle osteotomy (TTO) which distalizes the insertion of the tendon [21]. Patella tendon excision and repair has also previously been described to correct patella alta, but only in patients with cerebral palsy who have crouch gait deformity [22]. In this case report, we decided to proceed with the more conservative of these two patellar advancement options for several reasons; a TTO procedure is a particularly aggressive procedure [23] more commonly performed for the indication of patellar instability [21], persistent symptoms are reported in the majority of patients receiving a TTO [21], and it was also believed that this patient's tendon had stretched from the site of the index repair procedure thus compromising the integrity of this tissue. Excision of this portion of tendon followed by primary repair with suture anchors was the most appropriate approach in this case, especially after thinning of the proximal patellar tendon and obvious redundancy were confirmed intraoperatively.

The goals of this revision procedure were to restore functionality so that this patient may achieve his ADLs and return to work at some capacity. This was a very complex case, and the authors contend that the treatment approach was appropriate as it provided him with the greatest opportunity for a positive outcome. This was a salvage procedure, and as such it is satisfactory that he was able to regain ambulatory function with minimal pain. Alternative treatment options that could have been considered include augmentation of the index procedure, prolonged immobilization after the index procedure to prevent tendon lengthening and gap formation, conservative management rather than a revision procedure, or augmentation of the second repair following tendon resection. Patellar tendon resection followed by primary repair with PEEK corkscrew anchors was able to provide symptomatic relief with modest functional recovery in this patient with a failed patellar tendon repair.

## Figures and Tables

**Table 1:** Patient-reported outcomes.

PRO	Preop	Postop	Change
IKDC	27.6	33.3	5.7
KOOS			
Daily living	39.7	57.4	17.7
Pain	33.3	44.4	11.1
Physical symptoms	51.2	48.5	−2.7
Quality of life	12.5	25	12.5
Sports and recreation	5	5	0
Symptoms	35.7	39.3	3.6
JR	36.9	50	13.1
SF-12 mental	44.4	50.9	6.5
SF-12 physical	20	23	3
VR-12 mental	47.1	49.3	2.2
VR-12 physical	19.4	23.9	4.5
